# A self-management program for employees with complaints of the arm, neck, or shoulder (CANS): study protocol for a randomized controlled trial

**DOI:** 10.1186/1745-6215-14-258

**Published:** 2013-08-16

**Authors:** Nathan Hutting, J Bart Staal, Yvonne F Heerkens, Josephine A Engels, Maria WG Nijhuis-van der Sanden

**Affiliations:** 1 Scientific Institute for Quality of Healthcare, Radboud University Nijmegen Medical Centre, Nijmegen, The Netherlands; 2 Department of Occupation & Health, HAN University of Applied Sciences, P.O. Box 6960, 6503, Nijmegen, GL, The Netherlands; 3 Department of Physical Therapy, Radboud University Nijmegen Medical Centre, Nijmegen, The Netherlands

**Keywords:** CANS, RSI, RCT, Self-management, ehealth

## Abstract

**Background:**

Complaints of the arm, neck, or shoulder (CANS) have a multifactorial origin and cause considerable work problems, including decreased work productivity, sickness absence, and, ultimately, job loss. There is a need for intervention programs for people with CANS. Self-management is an approach used in chronic disease care to improve self-efficacy and wellness behaviors to facilitate participants to make informed choices and carry them out. This study will evaluate the effectiveness of a self-management program (including ehealth) and compare it to usual care among employees with chronic CANS (lasting >3 months).

**Methods/design:**

This is a randomized controlled trial in which 142 participants will be recruited and randomized (with pre-stratification) to either the intervention group (IG) or control group (CG). The IG will participate in a self-management program consisting of six group sessions and an ehealth module. The CG is allowed to use all usual care available. The primary outcome of the study is the self-reported disability of arm, shoulder, and hand, measured with the Disabilities of the Arm, Shoulder and Hand questionnaire (DASH). Secondary outcomes include: absenteeism, pain in the previous week, quality of life, catastrophizing pain, self-efficacy, workstyle, presenteeism, fatigue, the use of usual care, and limitations experienced on the job. Data are collected at baseline and at 3, 6, and 12 months follow-up.

**Discussion:**

Following the process of intervention mapping we developed a self-management program to suit and alleviate the problems and needs of employees with CANS. A strength of the study is that our intervention is specifically tailored to match the needs of employees with CANS. The study also has some potential weaknesses (for example, use of co-interventions, combination of group sessions and ehealth, self-reporting of data and possible contamination, Hawthorne effect, and recall or information bias) which are discussed.

**Trial registration:**

The trial is registered with the Dutch Trial Register (http://www.trialregister.nlNTR3816): (January 2013). The first participant was randomized in September 2012.

## Background

Complaints of the arm, neck, or shoulder (CANS) are common among people who work [1]. The reported point prevalence varies from 1.6% to 53% and the 12-month prevalence varies from 2.3% to 41% depending on the setting, definition, and classification used [2-4]. CANS is persistent; 77% of employees with CANS still have complaints after 6 months [5]. Also, about 19% of the patients report chronic complaints of which 58% report the use of healthcare, such as care given by the general practitioner, medical specialist, and physical therapist [3]. At one Dutch university, 11% of the employees reported regular physical complaints due to working with the computer and 4% reported to have these complaints very often [6].

Although musculoskeletal disorders of the upper extremity and neck are common, there is no international consensus on related terminology [7]. Terms such as ‘complaints of the arm, neck, or shoulder’ [8], ‘work-related upper extremity musculoskeletal disorders’ [9], ‘musculoskeletal upper extremity disorders’ [10], ‘neck and upper extremity complaints’ [5], ‘work-related upper limb disorders’ [11], and ‘repetitive strain injuries’ [12] are all frequently used. However, in these classifications a distinction is usually made between specific CANS (such as epicondylitis, carpal tunnel syndrome) and non-specific CANS [8]. The most recent consensus statement in the Netherlands was published in 2007 [13]. This multidisciplinary consensus on terminology was reached among healthcare professionals and supports the diagnosis and classification of all CANS not caused by acute trauma or by any systemic disease [8]. Within these complaints, 23 disorders are classified specific, because they were judged as diagnosable disorders by experts. All other complaints are labeled non-specific [8]. Non-specific CANS is characterized by pain or tingling sensations located in the arms, shoulders, neck, or upper back without a clear pathophysiological substrate [14]. Many people suffering from CANS have complaints in more than one region [3,5].

CANS causes major work problems, including presenteeism (decreased work productivity while at work), absenteeism (sickness absence), and, ultimately, job loss [1,15]. In the Netherlands, CANS is responsible for about 15% of the total number of sick days [11]. The total yearly costs in the Netherlands of neck and upper limb symptoms due to decreased productivity, sick leave, chronic disability for work, and medical costs has been estimated in 2003 at 2.1 billion Euros [7]. Thus, work-related neck and upper limb symptoms have both a medical and a substantial socioeconomic impact [16]. The annual prevalence of sickness absence due to work-related upper-extremity complaints is reported to be 2% to 4% of the general workforce [17]. Sickness absenteeism and permanent disability are important components of decreased productivity, but they represent only a part of its total cost [1]. A considerable proportion of health-related productivity loss derives from presenteeism, that is, decreased work performance while at work [1,18,19]. This is endorsed by Van den Heuvel et al. [19] who found that in 26% of the cases reporting CANS, productivity loss was involved. Moreover, in 68% of all cases reporting productivity loss, this was due to decreased productivity at work, while the other 32% was due to sickness absence [19].

Although the exact etiology of non-specific CANS is unknown, it is reported to be of multifactorial origin [20-23]. Physical characteristics (that is, wrong working posture, repetitive work), psychosocial characteristics (that is, lack of social support from colleagues or superior), personal factors (that is, an ineffective approach to stress management) of the individual worker as well as characteristics of their work environment (that is, high job demands, lack of control), contribute to the development and persistence of complaints [5,20-28]. The importance of each factor, and its individual contribution to the risk of provoking symptoms, varies among individuals and work environments [29].

Communication with supervisors to understand the needs and challenges of the employee is essential, and tailoring of an intervention to accommodate the employees’ needs is important [30]. Work-related factors (that is, high job strain) seem to be important determinants of perceived disability, especially among younger employees [1]. Favorable psychosocial work characteristics might prevent productivity loss in symptomatic employees with CANS [19]. Among office workers, an adverse work style increases the risk of having upper-extremity pain [29,31]. Because work style consists of a combination of factors (for example, working through pain, handling deadlines/pressure, handling stress, and self-imposed workload) interventions could be developed to address each of these individually [29]. Pain intensity and its impact on work and sleep, psychosocial factors (that is, lack of social support of colleagues or superior, work pressure), as well as physical factors at work (that is, wrong working posture) should all be included as potential targets for interventions to improve the management of disability caused by CANS [1,5,32].

Despite the multifactorial origin of CANS, most intervention studies focused only on the physical components of the workplace [16]. There is limited evidence on the effectiveness of exercises when compared to massage; adding breaks during computer work; massage as add-on treatment to manual therapy, and manual therapy as add-on therapy to exercises [33]. There is conflicting evidence concerning the effectiveness of exercises over no treatment or as add-on treatment, and no differences were found between various kinds of exercises [33]. Also, there is conflicting evidence regarding the effectiveness of ergonomic interventions [9,10,33-36].

Nowadays, multi-component interventions that include both biomechanical and psychosocial components are recommended [9,22,37]. Bernaards et al. [38,39] developed a workstyle intervention for computer workers, which focused on behavioral change with regard to body posture, workplace adjustment, breaks, and coping with high work demands. This intervention was effective in improving recovery from neck/shoulder symptoms and reducing pain in the long term (12 months) compared to usual care, whereas no effects were found after 6 months and in arm/wrist/hand pain [40].

There seems to be a need for intervention programs for people suffering from CANS [8,31]. Among Dutch employees with sickness absence due to CANS, 24% believe that work is mainly the cause of their complaints and 30% stated that these complaints are partly caused by work [40]. Also, 19% of the Dutch employees stated that measures at work are needed in the area of CANS because these are either not, or insufficiently, available. Self-management is an approach increasingly used in chronic disease care to improve self-efficacy and wellness behaviors [41]. Barlow et al. [42] defined self-management as ‘the ability to manage the symptoms, treatment, physical and psychosocial consequences, and lifestyle changes inherent in living with a chronic condition’. Self-management programs aim to help participants make informed choices and then carry them out [43]. Key self-management skills include: problem-solving, decision-making, resource utilization, forming partnerships with healthcare providers, and taking action [43]. Program participants are up-skilled in personalized goal setting and action care planning. Collaborative problem definition is based on their readiness to change and self-efficacy [44]. Self-management interventions focus primarily on encouraging patients to be involved with and in control of their own treatment, as well as improving their understanding of how their condition and treatment affect their lives [45]. As a result, self-management interventions reflect a change from a patient passively receiving care to a collaborative model in which the patient and provider share their knowledge and work together to achieve a goal of optimal self-management [45].

There is inconsistent evidence for the effects of self-management programs for patients with chronic musculoskeletal pain [46-48], and there is some evidence that group-delivered short programs (<8 weeks) with a healthcare professional involved have the best potential [46]. A multi-component pain and stress self-management group intervention was found to have better effect than individually administered physical therapy in the treatment of persistent musculoskeletal tension-type neck pain regarding coping with pain, in terms of patients’ self-reported pain control, self-efficacy, disability, and catastrophizing pain, over the 20-week follow-up [49].

Many employees suffering from CANS still go to work despite the feeling that, in view of their health, they should have taken sick leave [19]. Thus, employees with CANS continue working, which often results in the persistence of their complaints. In a study of Van Eijsden et al. [20] nearly all employees suffering from CANS said that they were very precise persons, and incapable of dealing with heavy workload or tight deadlines. Moreover, employees explained that they had high work standards and would ignore the workload and onset of symptoms, despite knowing that these symptoms could become chronic in a few months [20]. Thus, it seems important that employees suffering from CANS make informed choices and carry them out so that they have the ability to manage the symptoms, healthcare utilization, physical and psychosocial consequences, and lifestyle changes inherent to living with CANS. Self-management programs addressing physical characteristics, psychosocial characteristics, and personal factors of the individual worker, as well as characteristics of their work environment, may be useful for employees suffering from CANS.

Detaille et al. [50,51] developed a self-management program for employees in the Netherlands with a chronic disease. Following the process of intervention mapping [52,53] we adapted their program to suit and alleviate the problems and needs of employees suffering from CANS.

### Aim of the proposed study

This study will evaluate the effectiveness of a self-management program (including ehealth) compared to usual care, in employees suffering from chronic non-specific CANS (persisting >3 months).

## Methods/design

### Organization of the study

**Figure 1:**
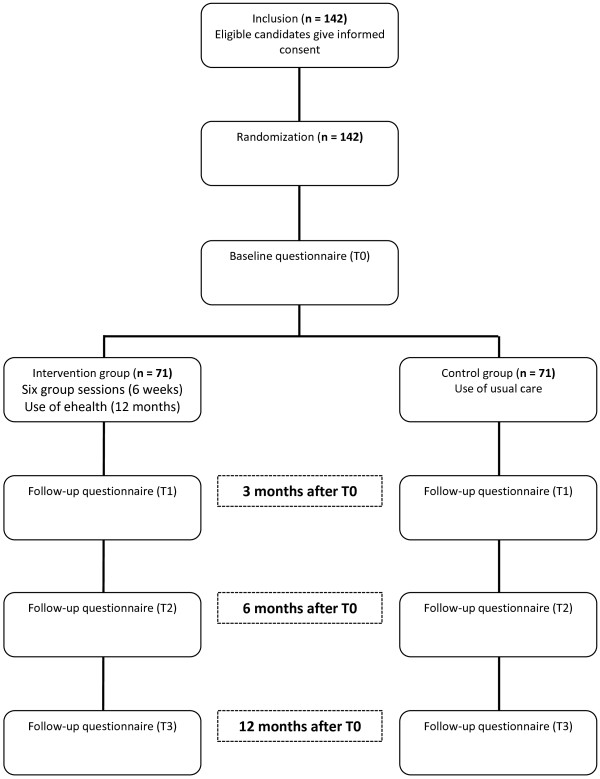
Flowchart showing the design of the trial.

This is a randomized controlled trial (RCT) with a follow-up of 1 year (Figure 1).

The Medical Ethics Committee (METC) of the Radboud University Nijmegen Medical Centre (RUNMC) approved the study design, protocols, and procedures. Participation is voluntary and participants can withdraw at any moment without any consequences. All participants will sign informed consent.

For the involved stakeholders the study is entitled: ‘Self-management and employability of workers with complaints of arm, neck, or shoulder; CANS sustainable under control’. For the potential participants the intervention is called ‘Control CANS’.

### Participants and recruitment

Participants will be recruited in two ways: (1) from employees of the RUNMC, the HAN University of Applied Sciences (HAN UAS) and Sanquin (Blood bank), all located in Nijmegen, the Netherlands. These potential participants will be recruited by newsletters within the organization and will be informed about the project by company physicians, occupational health coordinators and supervisors; and (2) recruitment of the general population in the area of Nijmegen. These potential participants will be recruited by calls in local newspapers and by contacting the Dutch patient group the ‘*RSI vereniging*’ (Association for Repetitive Strain Injury).

Candidates willing to participate can contact the first author (NH) who will arrange a consultation with a physical therapist. Eligible candidates will receive an information letter about the project at least 7 days before this consultation; this letter includes the information as approved by the METC as well as the informed consent letter. The physical therapist will provide additional information about the implications of participation. After this, the physical therapist will check the eligibility of the employee based on the inclusion and exclusion criteria (Table 1) by history-taking and screening of the employee (including filling out the Four-Dimensional Symptom Questionnaire (4DSQ) [54]). An extended version of exclusion criteria 1 and 4 is provided in Additional file 1. If considered necessary, the physical therapist will perform a brief physical examination to rule out any exclusion criterion. During this consultation each participant will be asked to sign informed consent. Furthermore, randomization will be performed. All data in the final publication of the trial, including the flow diagram of the progress of participants through the phases of the trial, will be reported according to the CONSORT 2010 statement [55].

**Table 1:** Eligibility criteria

**Inclusion criteria:**	
1.	Participant is in his/her opinion limited in performing his/her work (related to CANS)
2.	Participant suffers from work-related complaints
3.	Complaints must have persisted for at least 12 weeks (either a continuous or intermittent course)
4.	Participant works for at least 12 h a week
**Exclusion criteria:**	
1.	Red flags [36]^a^
2.	Complaints caused by a systemic disease [36]
3.	Complaints caused by traumatic injury [36]
4.	Suspicion of specific CANS [36]^a^
5.	Suspicion of mental pathology (>4 points on subscale depression, measured with the Four-Dimensional Symptom Questionnaire) [54]

^a^An extended version of exclusion criteria 1 and 4 is provided in Additional file 1.

### Randomization

Randomization to either the intervention group (IG) or control group (CG) will be performed at the patient level. Each participant is assigned to either the IG or CG by randomization with pre-stratification for the three participating companies and for participants from the general population (that is, four groups: RUNMC, HAN UAS, Sanquin, and the general population). The allocation sequence is computer-generated (http://www.randomization.com/). A researcher not involved in assigning participants to their groups will prepare concealed, consecutively numbered, sealed, opaque envelopes. Every envelope will contain a paper indicating the treatment allocation. Participants will receive their envelope during the consultation with the physical therapist who is not aware of the randomization sequence. Participants can open the envelope while with the physical therapist; subsequently, the physical therapist will inform the first author (NH) about the treatment allocation.

### Procedures

Participants allocated to the IG will receive information (from NH) about the next series of the self-management program after randomization. At the start of the program they also will receive the login code for the program website. Both the IG and CG will receive their baseline questionnaire (T0) in the week before the start of the self-management program of the IG. The other questionnaires will be provided at 3 months (T1), 6 months (T2), and 12 months (T3) after T0. All questionnaires will be provided digitally on a secured website.

### Interventions

#### Development

The self-management program described below was compiled based on the self-management program developed by Detaille et al. [50,51]. Based on a recent multidisciplinary guideline for non-specific CANS [36], relevant literature, focus group sessions with employees with CANS, and relevant experts, and following the process of intervention mapping [52,53], we adapted the program developed by Detaille et al. [50,51] to suit and alleviate the problems/needs of employees suffering from CANS. Intervention mapping is a tool for the planning and development of health promotion interventions [56]. It maps the path from recognition of a need or problem to the identification of a solution and describes the process of health promotion program development in six steps: (1) needs assessment; (2) definition of proximal program objectives based on scientific analyses of health problems and problem causing factors; (3) selection of theory-based intervention methods and practical strategies to change (determinants of) health-related behaviors; (4) production of the program components and production; (5) anticipation of program adoption, implementation, and sustainability; and (6) anticipation of process and effect evaluation [53,56]. Intervention mapping is more an iterative rather than a linear process; program planners move back and forth between tasks and steps [56]. Each step in the process is based on previous steps, and inattention to a particular step may lead to mistakes and inadequate decisions [56]. The complementary ehealth was also developed based on the recent multidisciplinary guideline for non-specific CANS [36], relevant literature, focus group sessions with employees suffering from CANS, and relevant experts.

#### Group sessions and ehealth

The program will consist of 6 weekly group sessions of 2.5 h each with four to 12 participants per group. The meetings are supervised by one or two trainers (depending on the group size). The first session will start with an introduction to the program and to the participants. Each subsequent session will start with summary reflection on the action plans made in the previous session. After this, the relevant topics will be discussed (Table 2). In one session an expert will give a presentation and will answer questions from the participants about exercises. At the end of each session participants will be asked to set targets (Specific, Measurable, Acceptable, Realistic, Time-bound [SMART], and formulated in terms of behavior) and action plans will be made.

**Table 2:** Topics of the group sessions and ehealth

**Topics of the group sessions:**	
*Session 1*	Introduction
	Dealing with a chronic disability
	Living with CANS
	Working with CANS
	Workload and work capacity
	What is self-management?
	Introduction to the ehealth module
*Session 2*	Discussion on the ehealth module
	Core qualities
	Time management
*Session 3*	Dealing with pain and fatigue
	Stress and stress management
	(Muscle) relaxation exercises
*Session 4*	Healthy lifestyle
	Nutrition
	Exercises and sports
	Use of facilities
*Session 5*	Communication skills
	Working with others and asking for help
*Session 6*	Dealing with negative emotions
	Positive thinking
	Making a mind map
**Topics of the ehealth module:**	
***Topic:***	***Content:***
*Use of ehealth*	Manual of the ehealth module
*Self-management*	Introduction to self-management
*CANS*	Non-specific CANS, specific CANS, symptoms, causes (workload and capacity, physical factors, psychosocial and personal factors, chronic pain, sensitization, self-tests, and screening tests), prognosis
*Possible solutions*	What can I do? (workplace, work pressure and work style, reduction of stress, sports, and specific exercises), facilities within organization, treatments
*About the group sessions*	Topics of the group sessions and manual
*Further reading*	Additional information and references to websites

The group sessions are complemented by an ehealth module. On a secure website the topics of the group training are discussed. Also, additional information is available on self-management and on specific topics of CANS, such as etiological factors (physical, psychological, and social [work] factors), prognostic factors, lifestyle factors, and other modifiable factors (Table 2). The ehealth is available for the IG up to 12 months after T0 (that is, up to T3).

#### Control group

The CG can use all usual care and information available within the organization of the participant. They are also allowed to use all care available outside of the organization.

### Baseline characterization of participants

Evaluation of baseline characteristics provides insight into the generalizability of the study, the success of the randomization, and any potential confounding factors. Measures regarding the baseline characteristics of the participants are provided in the list below.

Baseline characteristics of participants collected at T0:

– Gender

– Age (years)

– Weight, height

– Family situation

– Level of education

– Years of work experience

– Nature of employment

– Number of working days and hours

– Complaints related to CANS

– Dominant hand

– Duration of complaints

– Hours of computer work per day

– Assessment of repetitive work

– Alteration / variation / variety at work

– Working with elevated arms

– Working with rotated head

### Outcome assessment and data collection

#### Primary outcome

The primary outcome measure will be the self-reported disability (in the previous week) of arm, shoulder, and hand, measured with the Disabilities of the Arm, Shoulder and Hand questionnaire (DASH) [57]. The validity, test-retest reliability, and responsiveness of the DASH have been investigated extensively [57]. There is also preliminary evidence to support the use of DASH to measure upper extremity symptoms and disability in patients with neck pain. We will use the Dutch language version of the DASH (DASH-DLV) which is proven a reliable and valid instrument for assessing disability and symptoms in Dutch patients with a variety of unilateral upper limb disorders [58]. The DASH will be used at baseline (T0), and at 3 (T1), 6 (T2), and 12 months (T3) follow-up.

#### Secondary outcomes

Absenteeism will be measured by asking the participants the number of days they had been off work for all illnesses, as well as specifically for CANS, during the past 3 months (T1 and T2) or 6 months (T3). Presenteeism will be measured with the Stanford Presenteeism Scale (SPS-6) which has shown high internal consistency and good validity [59]. The Dutch version of the SPS-6 has shown good reliability [60] and the structural and discriminative validity of the Dutch version of the SPS-6 are also endorsed [60]. Presenteeism will also be measured with the Work Limitations Questionnaire which has demonstrated high reliability and validity [61].

Pain in the previous week will be measured with the Numeric Pain Rating Scale (NPRS, 0–10). Numeric pain scales are established as reliable and valid [62-64]. The NPRS showed good test-retest reliability and responsiveness in patients with shoulder pain [65] and fair to moderate test-retest reliability in patients with mechanical neck pain [66]. Pain catastrophizing will be measured with the Pain Catastrophizing Scale (PCS) (Dutch language version) originally developed by Sullivan et al. [67] The validity of the PCS has been confirmed [68,69] and the PCS showed sufficient test-retest stability [70].

Self-efficacy will be measured with the Dutch Adaptation of the General Self-Efficacy Scale [71]. The General Self-Efficacy Scale is a 10-item psychometric scale designed to assess optimistic self-beliefs to cope with a variety of difficult demands in life (http://userpage.fu-berlin.de/health/selfscal.htm). The self-efficacy at work scale, developed by Detaille et al. [51], will be used to measure self-efficacy at work. The self-efficacy at work scale has shown high internal consistency (alpha 0.8) [51].

Fatigue will be measured with the Checklist Individual Strength which has shown good reliability and validity [72]. Burnout features will be measured with the Utrecht Burnout Scale (UBOS) [73], which is an adapted version of the Maslach Burnout Inventory [74]. The validity of the UBOS has been confirmed [75].

Work style will be measured with the Workstyle Short Form which has demonstrated acceptable psychometric properties [76]. Pace and amount of work, relations with colleagues/supervisors, need for recovery, and participation and empowerment on the workplace, will be measured by subscales of the Questionnaire on experiencing and assessing stress at work (VBBA; a questionnaire on experiencing/assessing stress at work) which is frequently used in the Netherlands to measure employees’ experience and evaluation of their work in the organization; the VBBA has shown to be a valid and reliable questionnaire [77].

Quality of life will be measured with the Dutch language version of the SF-12v2 [78]. The reliability and validity of the SF-12 have been confirmed [78]. Self-reflection and insight are measured with the Self-Reflection and Insight Scale which is a valid and reliable measure of self-reflection and insight [79].

Participants will also be inquired with self-developed questionnaires about the use of healthcare interventions (treatments, workplace adaptations, and conversations with supervisor) during the intervention and follow-up period, participation in sport (intensity and frequency), limitations experienced in work-related activities (limitations experienced in the previous 2 weeks, numeric rating scale (NRS), 0–10), work capacity (NRS, 0–10), working with complaints (opinion about working with complaints and number of working days working with complaints), and about self-management skills at work [51] (opinion about self-management skills at work, six-point Likert scale).

#### Process evaluation

A process evaluation will be performed to examine experiences of the participants with the intervention program. The process evaluation will be carried out using both quantitative and qualitative techniques. The experiences of the participants in the IG will be examined with a short questionnaire conducted at T3. Also, about 30 participants will be interviewed about their experiences with the program within 2 to 4 weeks after finishing the group sessions. The information obtained from these interviews can provide valuable information about the participants’ experiences with the program and can be used in treatment (programs) for employees with CANS.

### Sample size

The sample size calculation is based on the primary outcome of this study, the DASH. We assume that a difference in the score of 10 points on the DASH at T3 is a clinically relevant difference [80]. We used a standard deviation (SD) of 16.65 (based on the mean SD used by Bron et al. [80], Gummesson et al. [81], and van Eijsden-Besseling et al. [82]). Power analysis revealed a sample size of 71 participants in each group, assuming a dropout rate of 20%. This implies that a total of 142 patients will be needed. The difference in score on the DASH can be detected with a power of 0.90 and an alpha of 0.05.

### Blinding

Participants, trainers, and researchers cannot be blinded for the allocated treatment after randomization. However, all participants fill in the digital questionnaires at their home or at work, implying that the influence of researchers is ruled out. The data will be collected by the developer of the digital questionnaire, which will provide anonymous data to the researcher. Therefore, the analysis of the data by the researcher will be blind.

### Co-interventions

Participants of the IG are allowed to attend additional treatments (co-interventions). Information on all co-interventions received by participants in both the IG and the CG group will be collected by questionnaires at T3.

### Statistical analyses

Demographics will be presented in means and SD or median and interquartile ranges per group.

All outcomes of the questionnaires will be compared at baseline and at follow-up measurements. All analyses will be performed according to the intention-to-treat principle.

Longitudinal regression analysis will be performed to evaluate the effects of the intervention. For every outcome, the follow-up times (T1, T2, T3) will be defined as dependent variable whereas the difference in baseline values (T0) of the two groups will be defined as independent variable. Correction of confounders will be applied, if necessary.

To assess whether protocol deviations have caused bias, the results of the intention-to-treat analyses will be compared to per-protocol analyses. All data of the measurements used in this study will be provided digitally in an external system. All analyses will be performed with IBM SPSS Statistics 20.

### Ethics

This study protocol is approved by the Medical Ethics Committee of RUNMC (2012/319) and registered in the Dutch Trial Register (NTR3816) (http://www.trialgegister.nl).

Potential participants from outside the three participating organizations will receive a travel allowance of 0.19 eurocents per kilometer for attending the appointment with the physical therapist.

## Discussion

We present a study which aims to evaluate the effectiveness of a self-management program (including ehealth) and compare this with usual care in employees suffering from chronic non-specific CANS (persisting >3 months) using an RCT design.

Based on a recent multidisciplinary guideline [36], relevant literature, focus group sessions with employees suffering from CANS, and relevant experts, we adapted a self-management program for employees with a chronic disease in the Netherlands developed by Detaille et al. [50] to suit and alleviate the problems and needs of employees with CANS. The strength of this intervention is that the program is specifically tailored to the needs of employees with CANS. Focus group sessions with employees identified the needs of participants related to disease-specific information, exercises, muscle relaxation, working with pain, work and social environment, and personal factors (including work style).

To meet these needs we will combine the adapted self-management program with an additional ehealth component, which provides the participants with valuable information related to CANS. Participants are also up-skilled in personalized goal-setting and action-planning, and collaborative problem definition is based on their readiness to change and their self-efficacy. By making action plans, we expect participants to take actions to manage their symptoms, treatment, physical and psychosocial consequences, and lifestyle changes inherent to living with CANS. Strengths of the study include the RCT design and the use of pre-stratification, which excludes the chance of possible confounding by indication.

The study also has some potential weaknesses. One of them is the differential use of co-interventions in the groups. At T3 we will monitor the use of co-interventions in both groups, so that we can use this information when drawing our conclusions. We will also ask the IG whether the intervention has contributed to the use of co-interventions. There is a small risk of contamination because (in theory) it is possible that two direct colleagues will participate, albeit allocated to different groups; however, we consider this a small chance, since the three organizations involved have large numbers of employees (200–9,000 employees). Another potential weakness is that the questionnaires used in this study will be provided digitally. Although questionnaires will be provided only four times during the entire study, for employees with CANS doing computer work this might represent an extra load. However, since the questionnaires can be completed while taking breaks, we believe that participants are likely to fill in all the questionnaires.

We adapted our intervention based on the needs of employees of two participating organizations (HAN UAS and RUNMC). Based on the literature, we assume that their experiences and needs are largely the same as that of employees working in other institutes or companies. Moreover, we also adapted our self-management program based on focus group sessions with experts and on the literature. Therefore, we expect the program to be suitable for the general population of employees suffering from CANS. We realize that our program will demand some time and effort from the participants and that employees usually have little time to spare. Therefore, potential participants must be sufficiently motivated.

Although one strength of the study might be the combination of a self-management program and ehealth, this also implies that if the intervention is effective we do not know to what extent each component has contributed to these effects. Therefore, in the quantitative evaluation at T3 as well as in the qualitative evaluation of the program (in-depth interviews), we will ask participants about their experiences and the usefulness of both components.

The internal validity of this RCT might be affected by the fact that blinding of the participants is not possible. A potential source of bias is the difference in the amount of attention patients receive, also called the Hawthorne effect [83]. In our study, patients in the IG will participate in the group sessions, which might lead to an overestimation of the effect of the intervention program. Although in this study the CG is allowed to use usual care, which might lower the difference in attention between IG and CG, effects of the Hawthorne affect cannot be ruled out since participants are not blinded.

A final limitation might be that all data are self-reported. Therefore, as far as possible, we will use validated questionnaires. No objective data will be collected concerning absenteeism. There is ongoing discussion as to whether self-reported data on absenteeism are reliable, although recent evidence showed a high level of similarity between self-reported data and data from, for example, a national insurance authority, and that the use of self-reported data is justified in research [84]. There is also a possibility of recall or information bias. However, because this study is designed as an RCT we expect recall bias to occur to the same extent in both study groups.

## Trial status

Recruitment started in September 2012: inclusion is estimated to be finished in December 2013.

## Abbreviations

4DSQ: Four-Dimensional Symptom Questionnaire; CANS: Complaints of the arm, neck, or shoulder; CG: Control group; DASH: Disabilities of the Arm, Shoulder and Hand questionnaire; DASH-DLV: Dutch language version of the DASH; HAN UAS: HAN University of Applied Sciences; IG: Intervention group; METC: Medical Ethics Committee; NPRS: Numeric Pain Rating Scale; NRS: Numeric rating scale; PCS: Pain Catastrophizing Scale; RCT: Randomized controlled trial; RSI: Repetitive strain injury; RUNMC: Radboud University Nijmegen Medical Centre; SD: Standard deviation; SMART: Specific, measurable, acceptable, relevant, time-bound; SPS-6: Stanford Presenteeism Scale; UBOS: Utrecht Burnout Scale; VBBA: Questionnaire on experiencing and assessing stress at work.

## Competing interests

The authors declare that they have no competing interests.

## Authors’ contributions

NH participated in the final design of the study, developing the study protocol, and drafted the manuscript. JBS participated in the development of the initial study proposal, acquiring funding, the final design of the study, developing the study protocol, and commented on the draft versions of the manuscript. YH participated in the development of the initial study proposal, acquiring funding, the final design of the study, developing the study protocol, and commented on the draft versions of the manuscript. JE participated in the development of the initial study proposal, acquiring funding, the final design of the study, developing the study protocol, and commented on the draft versions of the manuscript. MN participated in the development of the initial study proposal, acquiring funding, the final design of the study, developing the study protocol, and commented on the draft versions of the manuscript. All authors read and approved the final manuscript.

## Supplementary Material

Additional file 1 **Extended version of exclusion criteria 1 and 4 **[36]**.** Red Flags: General slump, unintentional weight loss, fever, night sweats, non-mechanic pain, neuropathic pain, neurological symptoms (muscle weakness, isolated atrophy, radicular failure symptoms), signs of inflammation, history of malignancy, dyspnea, chest pain. Suspicion of specific CANS: radicular symptoms (severe radiating pain), shoulder pain with general loss of both active and passive movement (Capsulitis Adhaesiva), loss of muscle strength, symptoms of nerve stimulation, local pain combined with swelling or redness, difficulties to bending (pain) or stretching of a finger or thumb, typical palmar nodules, especially in the 4th and 5th finger, flexion contracture at the level of the MCP and PIP joints (Morbus Dupuytren), persistent joint pain that increases with stress on joints, age >44 years, mild transient morning stiffness and benign thickening especially in PIP joint (Bouchard’s nodes) and DIP joints (nodules of Heberden) (osteoarthritis). Click here for file
