# Supplementary material for: A self-management program for employees with complaints of the arm, neck, or shoulder (CANS): study protocol for a randomized controlled trial
Source: Trials. 2013 Aug 16;14:258. doi: 10.1186/1745-6215-14-258 (PMC3751728; doi:10.1186/1745-6215-14-258)
Supplement: Additional file 1 — Extended version of exclusion criteria 1 and 4 [36]. Red Flags: General slump, unintentional weight loss, fever, night sweats, non-mechanic pain, neuropathic pain, neurological symptoms (muscle weakness, isolated atrophy, radicular failure symptoms), signs of inflammation, history of malignancy, dyspnea, chest pain. Suspicion of specific CANS: radicular symptoms (severe radiating pain), shoulder pain with general loss of both active and passive movement (Capsulitis Adhaesiva), loss of muscle strength, symptoms of nerve stimulation, local pain combined with swelling or redness, difficulties to bending (pain) or stretching of a finger or thumb, typical palmar nodules, especially in the 4th and 5th finger, flexion contracture at the level of the MCP and PIP joints (Morbus Dupuytren), persistent joint pain that increases with stress on joints, age >44 years, mild transient morning stiffness and benign thickening especially in PIP joint (Bouchard’s nodes) and DIP joints (nodules of Heberden) (osteoarthritis). [file 1745-6215-14-258-S1.doc]

Additional file

Appendix 1: Extended version of exclusion criteria 1 and 4 .

Red Flags:

- General slump
- Unintentional weight loss
- Fever, night sweats
- Non-mechanic pain
- Neuropathic pain
- Neurological symptoms (muscle weakness, isolated atrophy, radicular failure symptoms)
- Signs of inflammation
- History of malignancy
- Dyspnea, chest pain

**Suspicion of specific CANS:**

- Radicular symptoms (severe radiating pain)
- Shoulder pain with general loss of both active and passive movement (Capsulitis Adhaesiva)
- Loss of muscle strength
- Symptoms of nerve stimulation
- Local pain combined with swelling or redness
- Difficulties to bending (pain) or stretching of a finger or thumb
- Typical palmar nodules, especially in the 4th and 5th finger, flexion contracture at the level of the MCP and PIP joints (Morbus Dupuytren)
- Persistent joint pain that increases with stress on joints, age > 44 years, mild transient morning stiffness and benign thickening especially in PIP joint (Bouchard's nodes) and DIP joints (nodules of Heberden) (osteoarthritis).
